# New perspectives on the molecular mechanisms of stress signalling by the nucleotide guanosine tetraphosphate (ppGpp), an emerging regulator of photosynthesis in plants and algae

**DOI:** 10.1111/nph.18604

**Published:** 2022-12-02

**Authors:** Marwa Mehrez, Shanna Romand, Ben Field

**Affiliations:** ^1^ Aix‐Marseille University, CEA, CNRS, BIAM, UMR7265 13009 Marseille France; ^2^ Faculty of Sciences of Tunis, Laboratory of Molecular Genetics, Immunology and Biotechnology University of Tunis El Manar 2092 Tunis Tunisia

**Keywords:** algae, chloroplast, guanosine tetraphosphate, nitrogen, photosynthesis, ppGpp, RelA SpoT Homologue

## Abstract

The nucleotides guanosine tetraphosphate and guanosine pentaphosphate (together (p)ppGpp) are found in a wide range of prokaryotic and eukaryotic organisms where they are associated with stress signalling. In this review, we will discuss recent research highlighting the role of (p)ppGpp signalling as a conserved regulator of photosynthetic activity in the chloroplasts of plants and algae, and the latest discoveries that open up new perspectives on the emerging roles of (p)ppGpp in acclimation to environmental stress. We explore how rapid advances in the study of (p)ppGpp signalling in prokaryotes are now revealing large gaps in our understanding of the molecular mechanisms of signalling by (p)ppGpp and related nucleotides in plants and algae. Filling in these gaps is likely to lead to the discovery of conserved as well as new plant‐ and algal‐specific (p)ppGpp signalling mechanisms that will offer new insights into the taming of the chloroplast and the regulation of stress tolerance.

**Table:** 

	Contents	
	Summary	1086
I.	Introduction	1086
II.	Specialisation of RSH enzymes for (p)ppGpp metabolism in plants and algae	1088
III.	The physiological roles of (p)ppGpp in photosynthetic eukaryotes	1088
IV.	Molecular mechanisms of (p)ppGpp signalling in plants and algae	1091
V.	Concluding remarks	1096
	Acknowledgements	1096
	References	1096

## I. Introduction

**Fig. 1:**
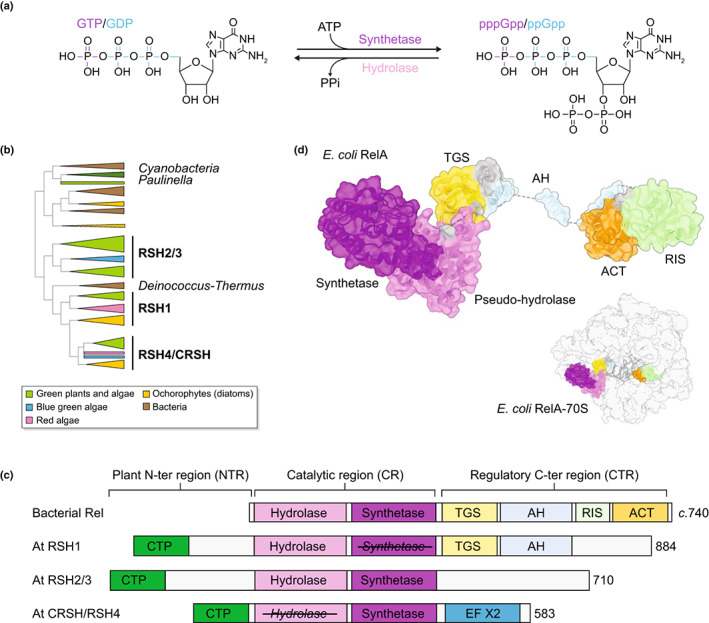
RelA SpoT Homologue (RSH) enzymes are involved in guanosine tetraphosphate (ppGpp) and pentaphosphate (pppGpp) biosynthesis in plants, algae and bacteria. (a) Outline of the synthesis and hydrolysis of (p)ppGpp by RSH superfamily enzymes. (b) A schematic outline showing the evolutionary relationship of RSH enzymes based on the phylogenetic analysis of Avilan *et al*. (2019). The three main plant and algal families are shown (RSH1, RSH2/3 and RSH4/CRSH). (c) The domain structure of long RSH in bacteria and Arabidopsis. For bacteria, we show the structure of a typical member of the Rel subgroup which is thought to represent the original bacterial long RSH (Atkinson *et al*., 2011). (d) The structure of *Escherichia coli* RelA, a bacterial long RSH, when bound to the ribosome. Inset shows the position of RelA on the ribosome, and the uncharged tRNA (grey) that interacts with RelA in the AH domain of the regulatory C‐terminal region. Protein Data Bank identifier, 5L3P. CTP, chloroplast transit peptide; hydrolase, (p)ppGpp hydrolase domain; synthetase, (p)ppGpp synthetase domain; TGS, ThrRS, GTPase, and SpoT; AH, alpha‐helical domain; RIS, ribosome‐intersubunit domain; ACT, aspartate kinase–chorismate mutase–tyrA (prephenate dehydrogenase); EF X2, two EF‐hand domains. Crossed‐out text indicates the presence of a domain that is not catalytically active.

Thanks to the domestication of the chloroplast, plants and algae are among the most successful and important organisms on the planet. A pair of purine nucleotides called guanosine tetraphosphate (ppGpp) and guanosine pentaphosphate (pppGpp), collectively (p)ppGpp, may have helped tame the chloroplast and at the same time allow efficient acclimation to environmental fluctuations. These signalling molecules (also known as alarmones), which are synthesised from GTP/GDP and ATP by RelA SpoT Homologue (RSH) enzymes (Fig. 1a), were discovered > 50 yr ago in bacteria where they play a major role in growth control and in acclimation to environmental change by targeting a wide range of effector enzymes to slow proliferation and promote resilience (Irving *et al*., 2020; Bange *et al*., 2021). (p)ppGpp signalling (also referred to as the stringent response) was discovered more recently in plants and algae (van der Biezen *et al*., 2000; Takahashi *et al*., 2004), where it takes place in the chloroplast (Boniecka *et al*., 2017; Field, 2018). The chloroplast is the site of photosynthesis, which fuels plant growth and nearly all life on earth by converting sunlight into chemical energy, and is also a hub for stress perception and regulation (Kleine *et al*., 2021). (p)ppGpp signalling is therefore well placed to play important roles in regulating both the nutrition and stress acclimation of photosynthetic eukaryotes. Indeed, as we will discuss, recent studies highlight (p)ppGpp as a conserved regulator of photosynthetic activity and open new perspectives on the emerging roles of (p)ppGpp in acclimation to environmental stress. We will then look at how rapid advances in the study of prokaryotic (p)ppGpp signalling are now revealing gaps in our understanding of the molecular mechanisms of (p)ppGpp signalling in plants and algae. Filling in these gaps is likely to lead to the discovery of highly conserved mechanisms as well as new plant‐ and algal‐specific mechanisms that will offer fresh insights into the remarkable success of the cohabitation between the chloroplast and the eukaryotic cell, and a greater understanding of stress acclimation in these organisms.

## II. Specialisation of RSH enzymes for (p)ppGpp metabolism in plants and algae

RSH superfamily enzymes carry out the synthesis and hydrolysis of (p)ppGpp (Fig. 1a). A synthetase domain catalyses the Mg^2+^‐dependent transfer of a pyrophosphate group from ATP to the ribose 3′‐OH of GDP (or GTP) to form (p)ppGpp, whereas a hydrolase domain catalyses the Mn^2+^‐dependent removal of the 3′‐diphosphate from (p)ppGpp to produce GDP (or GTP) and pyrophosphate. Multi‐domain long RSH enzymes that possess both (p)ppGpp synthetase and hydrolase domains, as well as the related single‐domain small alarmone synthetases (SAS) and small alarmone hydrolases, have been identified in almost all bacterial groups studied, as well as in photosynthetic eukaryotes, and some members of the Archaea (Atkinson *et al*., 2011; Ito *et al*., 2017; Avilan *et al*., 2019). An interesting recent development is the identification of the SAS called Metazoan SpoT homologue 1 (MESH1) in animals, along with the presence of (p)ppGpp (Sun *et al*., 2010; Young *et al*., 2021; Ito *et al*., 2022). However, the absence of an obvious enzyme responsible for (p)ppGpp synthetase activity and the dual activities of MESH1 as an efficient hydrolase of both (p)ppGpp and NADPH mean that the physiological role played by (p)ppGpp in animals is not yet fully resolved (Mestre *et al*., 2022). Plants and algae have at least three conserved families of long RSH enzymes, RSH1, RSH2/3 and RSH4 (or Ca^2+^‐activated RSH, CRSH) (Atkinson *et al*., 2011; Ito *et al*., 2017; Avilan *et al*., 2019) (Fig. 1b). These families originated at an early stage in the evolution of the Archaeplastida because representatives can be found in the three major lineages—green plants and algae (Viridiplantae), red algae (Rhodophyta) and blue‐green algae (Glaucophyta). Evolutionary inference in multiple studies places the Archaeplastida RSH families far from the cyanobacteria, the likely ancestors of the chloroplast, pointing to a complex evolutionary history that may not be possible to explain by simple vertical descent. Indeed, the RSH1 family groups with RSH from the Deinococcus–Thermus bacteria (Atkinson *et al*., 2011; Ito *et al*., 2017; Avilan *et al*., 2019), and there are signs of the more recent emergence of clades of diatom RSH that may have involved lateral gene transfer from bacteria (Avilan *et al*., 2019).

**Fig. 2:**
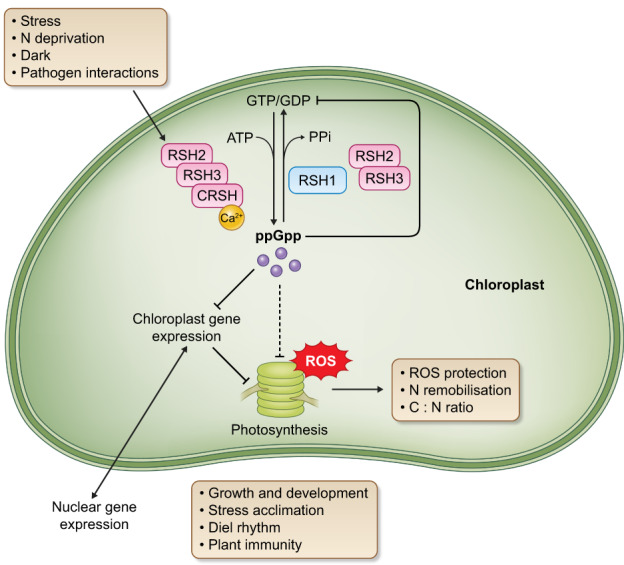
The physiological roles of guanosine tetraphosphate and guanosine pentaphosphate ((p)ppGpp) in *Arabidopsis thaliana*. A simplified outline of the known physiological roles of (p)ppGpp signalling in *Arabidopsis thaliana*. Dashed lines indicate potential interactions where there is no direct evidence, and blunt‐ended arrows indicate inhibition. ROS, reactive oxygen species.

Plant and algal RSH enzymes show important differences in domain structure from bacterial RSH, as well as a higher diversity and functional specialisation (Fig. 1c). The majority of plant and algal RSH so far tested are nuclear encoded and possess a predicted or experimentally verified chloroplast transit peptide (CTP). Except for the CTP and an N‐terminal extension, members of the RSH1 family show a strong resemblance to bacterial long RSH enzymes (Fig. 1c), with both (p)ppGpp hydrolase and synthetase domains, and a bacteria‐like C‐terminal regulatory region (CTR) with the threonyl‐tRNA synthetase‐GTPase‐SpoT and helical domains. While Arabidopsis lacks a clearly identifiable aspartate kinase–chorismate mutase–TyrA (prephenate dehydrogenase) domain in the CTR, this domain is found in the CTR of many other plant and algal RSH1 (Avilan *et al*., 2019). In bacteria, the CTR controls the switch between hydrolase and synthetase activities by interacting with partners such as stalled ribosomes (Fig. 1d) (Arenz *et al*., 2016; Brown *et al*., 2016; Loveland *et al*., 2016) and regulatory proteins (Battesti & Bouveret, 2006; Ronneau *et al*., 2019; Krüger *et al*., 2021). It is not yet known whether RSH1 family enzymes are also regulated by interactions at the CTR in a similar way. However, the report of an evolutionarily conserved interaction between Arabidopsis RSH1 and the chloroplastic ribosome‐associated GTPase spo0B‐associated GTP‐binding protein (ObgC) by a yeast two‐hybrid system suggests that such interactions are a real possibility (Bang *et al*., 2012). In the land plant‐clade of RSH1 enzymes, despite an early report showing ppGpp synthetase activity in *Escherichia coli* complementation assays (van der Biezen *et al*., 2000), it is now generally accepted that the synthetase domain is not catalytically functional (Mizusawa *et al*., 2008; Sugliani *et al*., 2016; Avilan *et al*., 2019), and the enzyme functions as the main (p)ppGpp hydrolase limiting (p)ppGpp levels *in planta* (Sugliani *et al*., 2016; Li *et al*., 2022) (Fig. 2).

RSH2/RSH3 family enzymes show bifunctional (p)ppGpp synthetase/hydrolase activity and in Arabidopsis act as the major ppGpp synthetases during the day (Maekawa *et al*., 2015; Sugliani *et al*., 2016) and are required for constraining (p)ppGpp levels at night (Ono *et al*., 2020). In addition to the catalytic region, plant RSH2/RSH3 enzymes have significant N‐terminal region (NTR) and CTR extensions with high sequence conservation, which bear little or no homology to their bacterial counterparts (Fig. 1c). The RSH2/RSH3 NTR and CTR may therefore be involved in novel, plant‐specific regulatory processes.

The RSH4/CRSH family enzymes identified so far all contain a nonfunctional (p)ppGpp hydrolase domain, and except for the CTP they lack an extension in the NTR. In plants and some green algae, the CTR contains EF‐hand domains which permit the Ca^2+^‐mediated activation of (p)ppGpp synthetase activity (Tozawa *et al*., 2007; Masuda *et al*., 2008a; Avilan *et al*., 2019) (Fig. 1c). Interestingly, the acquisition of novel domains is frequent among algal members of the RSH4 family (Ito *et al*., 2017; Avilan *et al*., 2019). This suggests that the regulation of synthetase domain activity observed in bacterial RSH can readily be repurposed to permit new regulatory connections.

## III. The physiological roles of (p)ppGpp in photosynthetic eukaryotes

Although the (p)ppGpp pathway was discovered some time ago in plants and algae, it is only recently that significant progress has been made in understanding its physiological roles. Progress has come principally from manipulating ppGpp levels *in vivo* (pppGpp is not usually detected in plants) via the use of different *RSH* mutants or the expression of ppGpp synthetases and hydrolases initially in Arabidopsis (Maekawa *et al*., 2015; Yamburenko *et al*., 2015; Sugliani *et al*., 2016; Abdelkefi *et al*., 2018; Honoki *et al*., 2018; Ono *et al*., 2020; Goto *et al*., 2022; Romand *et al*., 2022), and more recently using similar approaches in rice, moss and algae (Imamura *et al*., 2018; Avilan *et al*., 2021; Harchouni *et al*., 2022; Ito *et al*., 2022; Li *et al*., 2022). These studies have highlighted the role of (p)ppGpp signalling in regulating chloroplast function (and in particular photosynthesis) during growth and development, acclimation to nitrogen starvation, and the onset of night and immune responses (Fig. 2).

### 1. ppGpp is a conserved regulator of photosynthesis in plants and algae

A common theme emerging from multiple studies on plants and algae is that manipulation of ppGpp levels alters photosynthetic activity. Specifically, ppGpp accumulation causes a decrease in photosynthesis – reducing maximal quantum efficiency (*F*_v_/*F*_m_) and operating efficiency (or quantum yield) and electron transport rate (Sugliani *et al*., 2016; Honoki *et al*., 2018; Avilan *et al*., 2021; Harchouni *et al*., 2022; Ito *et al*., 2022; Li *et al*., 2022; Romand *et al*., 2022). In Arabidopsis, moss and diatoms, these changes are associated with modifications in the architecture of the photosynthetic electron transport chain. Notably at photosystem II (PSII), there is a decrease in the quantity of PSII reaction centres compared with the peripheral light‐harvesting antenna (Maekawa *et al*., 2015; Sugliani *et al*., 2016; Avilan *et al*., 2021; Harchouni *et al*., 2022). Photosystem I (PSI) would appear to be less affected, although more work is required to determine the relative impact of ppGpp on PSI and PSII. Rubisco levels also drop markedly in response to ppGpp accumulation, and *RSH* mutants deficient in (p)ppGpp metabolism show defects in nitrogen remobilisation from Rubisco during stress‐induced senescence (Maekawa *et al*., 2015; Sugliani *et al*., 2016; Honoki *et al*., 2018; Harchouni *et al*., 2022; Li *et al*., 2022; Romand *et al*., 2022). Interestingly, while the decrease in photosynthetic activity in response to ppGpp accumulation is conserved, certain features vary. For example, Rubisco is not sensitive to even very high levels of ppGpp in the diatom *Phaeodactylum tricornutum* (Avilan *et al*., 2021). This suggests that (p)ppGpp is able to trigger specific responses in different photosynthetic organisms.

The effects of ppGpp on photosynthesis were first established via the artificial overaccumulation of ppGpp. The relevance of these effects was also demonstrated at physiological levels of ppGpp in wild‐type (WT) organisms, as well as in the absence of stress (Sugliani *et al*., 2016; Romand *et al*., 2022). *RSH* mutants deficient in (p)ppGpp biosynthesis or hydrolysis show small defects in photosynthetic parameters under standard growth conditions (Sugliani *et al*., 2016; Honoki *et al*., 2018). More recently, very large ppGpp‐dependent effects on photosynthesis were observed during nitrogen starvation in Arabidopsis (Romand *et al*., 2022), and also in field‐grown rice plants carrying a mutation in the (p)ppGpp hydrolase gene *RSH1* (also known as *ABC1 REPRESSOR2*, *ARE2*) (Li *et al*., 2022). These points are discussed in detail in the following section.

### 2. (p)ppGpp signalling influences growth and development

Several studies have reported that the perturbation of (p)ppGpp levels has effects on growth and development. Such effects might be expected given the role of (p)ppGpp in the regulation of photosynthetic activity as discussed earlier. However, the situation is complex because (p)ppGpp overaccumulation in plants including Arabidopsis, rice and moss has variously been reported to increase (Maekawa *et al*., 2015), decrease (Sugliani *et al*., 2016; Li *et al*., 2022) or have no detectable effect on plant size (Harchouni *et al*., 2022; Ito *et al*., 2022). These conflicting results may be related to differing levels of (p)ppGpp, species‐specific effects, differences in light intensity/quality and nutrient levels. Indeed, there is recent support for the idea that variation in nutrient levels, which can vary considerably during the course of plant culture and in different growth substrates, might explain the different effects reported for ppGpp on plants size in Arabidopsis. Goto *et al*. (2022) showed that plants with high ppGpp levels can grow to a greater size than WT plants under moderately low nitrogen conditions. Defects in (p)ppGpp signalling also affect senescence. In Arabidopsis, dark‐induced and natural senescence are accelerated in mutants of the (p)ppGpp hydrolase RSH1 that have higher (p)ppGpp levels, and delayed in mutants unable to accumulate WT levels of (p)ppGpp (Sugliani *et al*., 2016; Li *et al*., 2022).

### 3. A pivotal role in acclimation to nitrogen deprivation

(p)ppGpp signalling was recently shown to play a major role in acclimation to nitrogen deprivation (Romand *et al*., 2022). The artificial accumulation of ppGpp in an *RSH3* overexpression line was found to protect Arabidopsis plants against nitrogen limitation (Maekawa *et al*., 2015; Honoki *et al*., 2018; Goto *et al*., 2022) suggesting that (p)ppGpp might be involved in acclimation. Using mutants defective in ppGpp accumulation, Romand *et al*. (2022) demonstrated that ppGpp accumulation is required for the acclimation of Arabidopsis plants to nitrogen limitation under physiological conditions that may be encountered in nature. Interestingly, another study performed in parallel further supports these findings by showing that the increase in ppGpp levels caused by a mutation in the rice (p)ppGpp hydrolase gene *RSH1* can suppress and overcome the constitutive nitrogen‐starved phenotype of the *ABNORMAL CYTOKININ RESPONSE1* mutant (Li *et al*., 2022). During nitrogen starvation, (p)ppGpp signalling is required for the safe downregulation of the photosynthetic machinery, whose products are no longer necessary due to a general growth arrest. ppGpp‐mediated downregulation of the photosynthetic machinery is associated with downregulation of chloroplast transcript levels, a reduction in the GTP pool, and remodelling of PSII (Romand *et al*., 2022). These changes are very similar to those observed in ppGpp‐overaccumulating lines (Maekawa *et al*., 2015; Sugliani *et al*., 2016). Limiting ppGpp biosynthesis during nitrogen starvation delays the downregulation of photosynthesis, and results in increased reactive oxygen species (ROS) accumulation, tissue damage, and a major disruption of the coordination between chloroplast and nuclear gene expression (Romand *et al*., 2022). Surprisingly, ppGpp levels do not increase to very high levels during nitrogen starvation, suggesting that (p)ppGpp signalling is somehow potentiated during stress by other factors. For example, potentiation of (p)ppGpp signalling could be related to the increase in the ppGpp/GTP ratio that occurs under nitrogen starvation, which would enhance the inhibition of enzymes where ppGpp is a competitive inhibitor. In any case, these findings show that the strong connection between (p)ppGpp signalling and photosynthesis is physiologically relevant and demonstrate a clear role for (p)ppGpp in abiotic stress acclimation. Downregulation of the photosynthetic machinery reduces carbon assimilation and at the same time liberates significant quantities of nitrogen as the machinery accounts for over half of leaf nitrogen in C3 plants (Evans & Clarke, 2019). While (p)ppGpp can reduce the risk of ROS accumulation from excessive photosynthetic activity during nitrogen limitation, it is likely that the nitrogen liberated from the photosynthetic machinery also serves a major role in supporting other cellular processes. Indeed, the photosynthetic and growth phenotypes of (p)ppGpp mutants under normal growth conditions (Sugliani *et al*., 2016; Honoki *et al*., 2018; Li *et al*., 2022) suggest that (p)ppGpp signalling continually fine‐tunes the cellular carbon/nitrogen equilibrium.

### 4. (p)ppGpp for quiet nights?

(p)ppGpp signalling is likely to be involved in regulating chloroplast gene expression at night in plants. In Arabidopsis, ppGpp levels increase at the onset of night (Ihara *et al*., 2015) in a CRSH‐dependent manner (Ono *et al*., 2020), although the final concentration requires the participation of RSH1, RSH2 and RSH3. The onset of night also triggers a transient Ca^2+^ flux into the chloroplast stroma (Johnson *et al*., 1995; Sai & Johnson, 2002) which may be responsible for directly activating CRSH via the EF‐hand domains in the CTR. While a *CRSH* mutant did not show any obvious growth phenotype, the authors observed a probable defect in the night‐triggered downregulation of transcript levels for certain chloroplast‐encoded genes (Ono *et al*., 2020). This may therefore be one of the processes by which dark‐induced stromal Ca^2+^ transients can influence chloroplast function (Rocha *et al*., 2014). Cyanobacteria and algae also accumulate (p)ppGpp in the dark but do not have RSH enzymes with EF‐hand domains for Ca^2+^ binding (Hood *et al*., 2016; Puszynska & O'Shea, 2017; Jin *et al*., 2022). This suggests that dark‐induced (p)ppGpp signalling is widespread in photosynthetic organisms, although the activation of RSH enzymes must occur via distinct mechanisms.

### 5. (p)ppGpp signalling influences plant immunity

The chloroplast, and in particular chloroplast‐generated ROS, plays a key role in plant immunity (Littlejohn *et al*., 2021). Therefore, it is perhaps not surprising that (p)ppGpp signalling, with its ability to downregulate photosynthesis where ROS are generated, can influence plant immunity against pathogens. The expression of plant *RSH2/3* genes is upregulated by plant pathogens, wounding, pathogen‐associated molecules and defence‐related hormones (Givens *et al*., 2004; Takahashi *et al*., 2004; Kim *et al*., 2009; Abdelkefi *et al*., 2018; Petrova *et al*., 2021). However, *RSH2/3* upregulation is associated with pathogen susceptibility suggesting that under at least some cases (p)ppGpp accumulation can favour the pathogen (Petrova *et al*., 2021). Consistent with this, overaccumulation of ppGpp in Arabidopsis leads to strong reductions in the levels of transcripts for defence‐related genes (Abdelkefi *et al*., 2018). Furthermore, high ppGpp levels lead to greater susceptibility to Turnip Mosaic virus, whereas lower levels are associated with increased resistance, accumulation of the defence hormone salicylic acid and precocious expression of the defence‐related protein PATHOGENESIS‐RELATED 1 (Abdelkefi *et al*., 2018). Pathogen‐associated molecular pattern (PAMP)‐triggered immunity (PTI) provokes stromal Ca^2+^ fluxes in a similar way to darkness, suggesting that CRSH might be activated during PTI. However, treatment of a *CRSH* mutant with the PAMP flagellin22 induced defence‐related genes in a similar fashion to the WT control (Ono *et al*., 2020). Therefore, the links between (p)ppGpp and Ca^2+^ signalling during immunity remain uncertain. Altogether, more work is required for understanding the full role that (p)ppGpp signalling has on plant immunity, and in particular during interactions with biotrophic and necrotrophic pathogens as well as herbivores. Furthermore, comparing the pathogenicity of WT pathogens with mutants unable to deliver their effector machinery may allow the identification of pathogens that are able to subvert (p)ppGpp signalling to overcome host defence.

## IV. Molecular mechanisms of (p)ppGpp signalling in plants and algae

Despite recent advances in understanding the physiological roles of (p)ppGpp as discussed earlier, few if any chloroplastic effectors of (p)ppGpp have been firmly identified. This contrasts with the situation in bacteria where (p)ppGpp and related nucleotides are known to interact directly with specific effector enzymes to regulate growth rate and promote stress acclimation and survival (Irving *et al*., 2020; Bange *et al*., 2021). Over recent years, the development of systematic approaches has led to a considerable expansion in the number of known (p)ppGpp‐binding proteins and effectors in bacteria. These advances were driven by techniques such as the differential radial capillary action of ligand assay, a rapid and quantitative method that can be used for testing candidate protein‐(p)ppGpp interactions in crude extracts and without the need for protein purification (Roelofs *et al*., 2011; Corrigan *et al*., 2016; Zhang *et al*., 2018), as well as by the use of ppGpp analogues to directly capture and identify ppGpp‐binding proteins in cellular extracts (Wang *et al*., 2019; Haas *et al*., 2022). Such approaches may also have considerable potential for identifying effectors in plants and algae. At the same time, the growing list of (p)ppGpp targets in bacteria also provides insights into the possible molecular mechanisms of (p)ppGpp signalling in plants. Indeed, bacterial ppGpp effectors are known today in transcription, nucleotide metabolism, translation, ribosome assembly, fatty acid biosynthesis and amino acid metabolism (Kanjee *et al*., 2012; Irving *et al*., 2020; Steinchen *et al*., 2020; Bange *et al*., 2021). Many of these processes are conserved in the chloroplasts of plants and algae and should therefore be considered potential targets of (p)ppGpp signalling (Table 1).

**Table 1:** Bacterial (p)ppGpp effectors and their likely chloroplastic orthologues in *Arabidopsis thaliana*.

Bacterial (p)ppGpp targets	Chloroplast orthologue(s)	*Escherichia coli*	*Bacillus subtilis*	Chloroplast	References
**Transcription**					
Core RNAP complex	PEP complex	*	–	*	Takahashi *et al*. (2004); Sato *et al*. (2009); Imamura *et al*. (2018)
Alpha1 (RpoA)	RpoA/AtCg00740				
Beta (RpoB)	RpoB/AtCg00190				
Beta′ (RpoC)	RpoC1/AtCg00180	*			Sato *et al*. (2009); Ross *et al*. (2013)
	RpoC2 /AtCg00170				
Omega (RpoZ)	No orthologue	*			Ross *et al*. (2013)
Sigma	Sig1/At1g64860, Sig2/At1g08540, Sig3/At3g53920, Sig4/At5g13730, Sig5/At5g24120, Sig6/At2g36990				
Transcription factor, DksA	no orthologue	*			Ross *et al*. (2016)
**Ribosome‐ and translation‐associated GTPases**					
Translation initiation factor 2 (IF2)	At1g17220/FUG1	*			Legault *et al*. (1972); Milon *et al*. (2006)
Elongation factor TU (EF‐TU)	At4g20360/SVR11	*			Legault *et al*. (1972); Rojas *et al*. (1984)
Elongation factor G (EF‐G)	At1g62750/SCO1, At1g45332, At2g45030	*			Rojas *et al*. (1984); Mitkevich *et al*. (2010)
Elongation factor 4 (EF4/LepA)	At5g08650/LepA	*			Zhang *et al*. (2018)
GTPase Der (Der/EngA)	At3g12080/Der	*			Bharat & Brown (2014)
GTPase Era (Era)	At5g66470/Era1	*			Corrigan *et al*. (2016); Zhang *et al*. (2018)
GTPase Obg (ObgE/CgtA)	At5g18570/ObgC	*	*		Buglino *et al*. (2002); Persky *et al*. (2009); Zhang *et al*. (2018)
GTPase HflX (Hflx)	At5g57960/HflX	*	*		Corrigan *et al*. (2016); Zhang *et al*. (2018)
GTPase BipA (BipA/TypA)	At5g13650/SVR3	*			Fan *et al*. (2015); Kumar *et al*. (2015)
GTPase RsgA (RsgA)	At1g67440/RsgA	*			Zhang *et al*. (2018)
GTPase RbgA (RbgA)	At4g02790/RbgA		*		Corrigan *et al*. (2016)
Translation release factor RF3	No orthologue	*			Kihira *et al*. (2012); Zhang *et al*. (2018)
**Purine metabolism**					
Adenylosuccinate synthetase (PurA)	At3G57610/ADSS	*			Stayton & Fromm (1979)
Amidophosphoribosyltransferase (PurF)	At2g16570/ASE1, At4g34740/ASE2, At4g38880/ASE3	*	–		Wang *et al*. (2019)
Inosine‐5′‐monophosphate dehydrogenase (GuaB)	At1g16350	(*)	*		Pao & Dyes (1981); Kriel *et al*. (2012); Wang *et al*. (2019)
Guanylate kinase (GmK)	At3g06200/GMK3/GKpm	–	*		Kriel *et al*. (2012); Liu *et al*. (2015); Nomura *et al*. (2014)
Hypoxanthine phosphoribosyltransferase (Hpt)	No chloroplast orthologue	*	*		Hochstadt‐Ozer & Cashel (1972); Kriel *et al*. (2012); Zhang *et al*. (2018); Anderson *et al*. (2019)
Xanthine phosphoribosyltransferase (XpT)	No orthologue		*		Anderson *et al*. (2020)
Adenine phosphoribosyltransferase (Apt)	No chloroplast orthologue	*	*		Hochstadt‐Ozer & Cashel (1972); Haas *et al*. (2022)
Nucleotide 5′‐monophosphate nucleosidase (YgdH/PpnN)	No orthologue	*			Zhang *et al*. (2018)
**Others**					
DNA primase (DnaG)	No orthologue	*			Wang *et al*. (2007); Maciag *et al*. (2010)
Lysine decarboxylase (LdcI)	No orthologue	*			Kanjee *et al*. (2011a)
Lysine decarboxylase (Ldcc)	No orthologue	*			Kanjee *et al*. (2011b)
Ornithine decarboxylase (SpeF)	No orthologue	*			Kanjee *et al*. (2011b)
Ornithine decarboxylase (SpeC)	No orthologue	*			Kanjee *et al*. (2011b)
pppGpp pyrophosphatase (GppA)	No chloroplast orthologue	*			Keasling *et al*. (1993)
(p)ppGpp synthetase (RelA)	At4g02260/RSH1, At3g14050/RSH2, At1g54130/RSH3, At3g17470/CRSH	*			Shyp *et al*. (2012); Zhang *et al*. (2018)
Hydrogenase maturation factor (HypB)	No orthologue	*			Zhang *et al*. (2018)
3‐Hydroxydecanoyl‐[acyl‐carrier‐protein] dehydratase (FabA)	No orthologue				Stein Jr. & Bloch (1976)
3‐Hydroxyacyl‐[acyl‐carrier‐protein] dehydratase (FabZ)	At2g22230, At5g10160				Stein Jr. & Bloch (1976)
Acetyl coenzyme A carboxylase (ACC)					Polakis *et al*. (1973)
Alpha subunit (AccA)	CAC3/At2g38040				
Beta subunit (AccD)	ACCD/AtCg00500				

A green square indicates the presence of a gene encoding the enzyme in the host genome. Lighter green indicates subunits of the same enzymatic complex. In the case of Arabidopsis, only chloroplast‐targeted (predicted or demonstrated) enzymes are shown. An asterisk indicates experimental evidence for (p)ppGpp binding, dashes indicate experimental evidence showing a lack of (p)ppGpp binding. Brackets indicate conflicting evidence. FabA, FabZ, ACC and Arabidopsis guanylate kinase are inhibited by (p)ppGpp but binding has not been directly shown. References for studies demonstrating inhibition, activation or binding of the indicated enzymes by (p)ppGpp are listed to the right.

### 1. Does (p)ppGpp directly inhibit chloroplast transcription?

Multiple studies show that (p)ppGpp accumulation *in vivo*, either artificially or during stress, results in the downregulation of chloroplast transcript levels in plants (Maekawa *et al*., 2015; Sugliani *et al*., 2016; Harchouni *et al*., 2022; Romand *et al*., 2022). Direct analysis of transcription by chloroplast run‐on or labelling of nascent transcripts in Arabidopsis indicates that the reduction in chloroplast transcript abundance caused by (p)ppGpp is due to the inhibition of transcription (Yamburenko *et al*., 2015; Sugliani *et al*., 2016). Chloroplast transcription is carried out by a bacterial‐like plastid‐encoded polymerase (PEP) and a phage‐like nucleus‐encoded polymerase (NEP). Some studies have observed a preferential effect of (p)ppGpp on the levels of PEP transcripts (Sato *et al*., 2009; Sugliani *et al*., 2016), while others have not observed a clear separation between NEP and PEP transcripts (Romand *et al*., 2022). The role of (p)ppGpp in regulating chloroplast transcription is therefore established; however, it is not yet clear exactly how (p)ppGpp is able to mediate this effect.

**Fig. 3:**
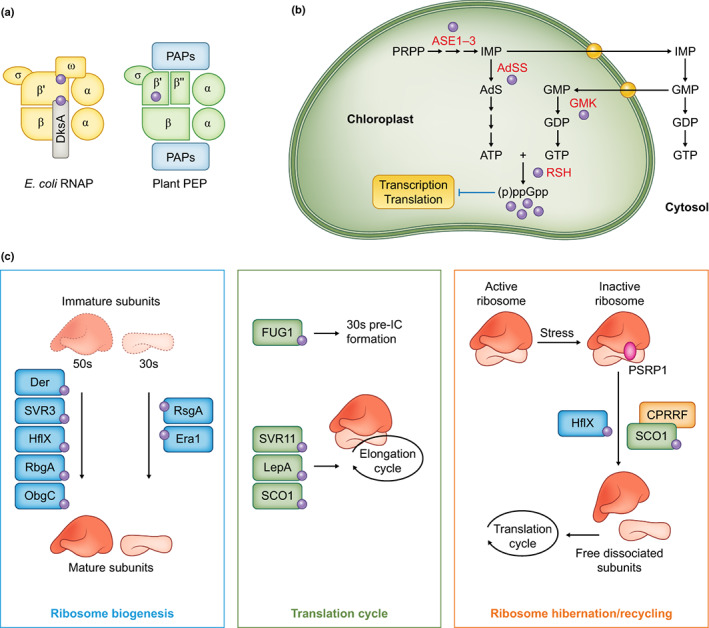
Known and potential targets of guanosine tetraphosphate and guanosine pentaphosphate ((p)ppGpp) in the chloroplast. (a) (p)ppGpp (purple circles) may be able to modulate chloroplast transcription through an interaction with the β′ subunit of the plastid encoded polymerase (PEP) (right). This interaction site is distinct to those found in *Escherichia coli* RNA polymerase (RNAP) (left). PAPs, PEP‐associated proteins. (b) Chloroplastic (p)ppGpp targets in purine metabolism. GmK is directly inhibited by (p)ppGpp *in vitro*, and other enzymes of purine metabolism are potential targets based on their predicted chloroplastic localisation and targeting by (p)ppGpp in bacteria. PRPP, phosphoribosylpyrophosphate; IMP, inosine monophosphate; AdS, adenylosuccinate. The blunt‐ended arrow indicates inhibition. (c) Chloroplastic enzymes implicated in ribosome biogenesis, translation and ribosome hibernation/recycling that may be inhibited by (p)ppGpp. PSRP1, plastid‐specific ribosomal protein 1 an orthologue of bacterial hibernation promoting factor (HPF). IC, initiation complex. See Table 1 for list of bacterial (p)ppGpp targets and their chloroplastic orthologues in plants.

In *E. coli*, (p)ppGpp directly modulates the activity of the RNA polymerase (RNAP) to downregulate the expression of ribosomal RNAs (rRNA) and upregulate the expression of genes involved in stress acclimation. The bacterial RNAP core is a complex composed of two α subunits, a β subunit, a β′ subunit, a ω subunit and a σ subunit (α_2_ββ'ωσ) (Fig. 3a; Table 1). There are two allosteric (p)ppGpp‐binding sites on RNAP that are conserved in *E. coli* and other proteobacteria. Site 1 is located at the interface between the β′ and ω subunits (Ross *et al*., 2013), and site 2 at the interface between the β′ subunit and the transcription factor DksA (Ross *et al*., 2016). The major transcriptional effects of (p)ppGpp accumulation in *E. coli* can be explained by (p)ppGpp binding at these two sites, although it does not explain all the effects of (p)ppGpp on growth (Wang *et al*., 2019). In plants, PEP has a bacterial‐like core complex consisting of two α subunits, a β subunit, β′ and β′′ subunits, and a σ subunit (α_2_ββ′β′′σ) (lgloi & Kössel, 1992; Suzuki *et al*., 2003; Borner *et al*., 2015) (Fig. 3a; Table 1). The β′ and β′′ subunits correspond to the N‐terminal and C‐terminal portions of the bacterial β′ subunit, and the same split is also present in the RNAP of cyanobacteria which is presumably ancestral. The plant PEP has also acquired additional co‐purifying accessory factors called PEP‐associated proteins that are not present in bacteria or even green algae (Pfannschmidt *et al*., 2000; Suzuki *et al*., 2004; Steiner *et al*., 2011). Strikingly, with regard to the action of (p)ppGpp, PEP completely lacks site 1 and site 2: there is no ω subunit, the conserved β′ K615 residue required for (p)ppGpp binding (Myers *et al*., 2020) is lacking from the corresponding Arabidopsis β′′ subunit, and there is no orthologue of the DksA transcription factor in plant or algal genomes (Fig. 3a; Table 1). From an evolutionary perspective, the lack of *E. coli*‐like (p)ppGpp‐binding sites is not surprising because the *E. coli* mechanism is a relatively recent evolutionary innovation that is restricted to the proteobacterial (Ross *et al*., 2016), and the RNAP of other bacterial groups is insensitive to (p)ppGpp.

Despite the clear absence of proteobacteria‐equivalent ppGpp‐binding sites for the control of PEP, several studies nevertheless indicate that PEP may be directly targeted by (p)ppGpp. Takahashi *et al*. (2004) first showed that exogenous application of ppGpp or pppGpp can inhibit transcription in chloroplast extracts. A follow‐on study then showed that ppGpp is able to specifically inhibit transcription in extracts enriched for PEP and not in extracts enriched for the alternative chloroplast RNAP NEP (Sato *et al*., 2009). Furthermore, radiolabelled 6‐thio‐ppGpp was found to bind to the PEP β′ subunit (Sato *et al*., 2009). It is therefore reasonable to suppose that a novel ppGpp‐binding site on the β′ subunit is necessary for PEP inhibition, although the exact residues involved remain to be identified and tested. ppGpp was also found to inhibit transcription of the chloroplast 16S rRNA in crude extracts from the unicellular red alga, *Cyanidioschyzon merolae* (Imamura *et al*., 2018). In both plants and algae, the concentration of ppGpp required for the *in vitro* inhibition of chloroplast transcription is at the high end of ppGpp sensitivities observed for bacterial enzymes (Steinchen *et al*., 2020), and is higher than the levels estimated to naturally occur within the chloroplast under nonstress conditions (*c*. 1–3 μM) (Ihara *et al*., 2015; Sugliani *et al*., 2016; Ito *et al*., 2022). The authors of both the plant and algal PEP studies therefore propose that, *in vivo*, other unidentified factors may potentiate the action of ppGpp on PEP in a similar manner to DksA (Sato *et al*., 2009; Imamura *et al*., 2018). The possibility of very local peaks in ppGpp concentration has also been suggested, and these findings could also point to the existence of strong and weak targets of (p)ppGpp in the chloroplast to allow for a graded response to (p)ppGpp levels as observed in bacteria (Steinchen *et al*., 2020).

### 2. Purine nucleotide metabolism, a universal target of (p)ppGpp signalling?

Purine biosynthesis has emerged as a major target of (p)ppGpp signalling in diverse bacteria (Irving *et al*., 2020; Bange *et al*., 2021). A large part of the purine biosynthetic pathway takes place in the chloroplast of plants and algae and involves orthologues of the bacterial enzymes (Fig. 3b; Table 1) (Smith & Atkins, 2002; Kusumi & Iba, 2014; Witte & Herde, 2020). These enzymes include orthologues of the bacterial (p)ppGpp targets adenylosuccinate synthetase (PurA or ADSS) (Stayton & Fromm, 1979; Wang *et al*., 2019; Yang *et al*., 2020a), amidophosphoribosyltransferase (PurF or ASE) (Wang *et al*., 2019), inosine‐5′‐monophosphate dehydrogenase (GuaB or IMDH) (Gallant *et al*., 1971; Kriel *et al*., 2012), guanylate kinase (GmK) (A. Kriel *et al*., 2012) and the RSH enzymes themselves (Steinchen *et al*., 2015; Zhang *et al*., 2018; Yang *et al*., 2020b). Currently, there is evidence that the chloroplast guanylate kinase of plants is inhibited at physiological ppGpp levels *in vitro* (Nomura *et al*., 2014). Furthermore, there is evidence that (p)ppGpp signalling can affect plant purine metabolism *in vivo* with the recent demonstration that (p)ppGpp accumulation is required to promote a decrease in total GTP levels during nitrogen starvation stress in Arabidopsis (Romand *et al*., 2022). In addition, overexpression of *RSH3* in the conditional GmK mutant *virescent‐2* in rice strongly enhanced the mutant phenotype, suggesting an interaction between increased ppGpp levels and reduced GmK function (Ito *et al*., 2022). However, the situation may be more complex than it appears because artificially increasing (p)ppGpp levels does not always affect the total GTP pool (Bartoli *et al*., 2020; Avilan *et al*., 2021).

The inhibition of bacterial purine nucleotide metabolism by (p)ppGpp may occur for several reasons. These include the conservation of metabolic precursors to allow a rapid return to growth, meeting the reduced demands of bulk RNA biosynthesis which itself is also a target of inhibition by (p)ppGpp, and reducing GTP levels to downregulate growth and potentiate the competitive inhibition of GTP‐dependent enzymes targeted by (p)ppGpp (Wang *et al*., 2020). Indeed, a (p)ppGpp‐mediated decrease in GTP is required for downregulation of transcription in the Firmicute, Actinobacteria and Deinococcus–Thermus groups of bacteria where RNAP is (p)ppGpp insensitive (Krasny & Gourse, 2004; Liu *et al*., 2015). In the model Firmicute *Bacillus subtilis*, (p)ppGpp accumulation causes a drop in GTP levels via the inhibition of guanylate kinase. This reduction in GTP levels inhibits transcription from genes where GTP is the initiating NTP, which notably includes the rRNA genes. A similar mechanism may explain the observed downregulation of chloroplast transcription by ppGpp in plants (Yamburenko *et al*., 2015; Sugliani *et al*., 2016). As discussed earlier, the chloroplastic GmK is specifically inhibited by ppGpp (Nomura *et al*., 2014), and GTP levels drop in a ppGpp‐dependent fashion under physiological stress conditions (Romand *et al*., 2022). GTP is also the initiating NTP for the chloroplast operon containing the 23S and 16S rRNAs in at least several plant species (Sugliani *et al*., 2016). Therefore, multiple lines of evidence point to the existence of a firmicute‐like mechanism for regulating transcription in chloroplasts. However, more detailed direct investigations into the role of GmK in plant (p)ppGpp signalling are required for demonstrating a direct causal link between ppGpp‐mediated inhibition of GmK and the inhibition of chloroplast transcription.

### 3. A role for ppGpp in regulating chloroplast translation?

In bacteria, (p)ppGpp signalling downregulates global translation by targeting a wide range of GTP‐binding enzymes involved in translation as well as in ribosome biogenesis and ribosome hibernation/recycling (Table 1) (Irving *et al*., 2020; Bange *et al*., 2021; Zegarra *et al*., 2023). (p)ppGpp is also likely to directly regulate translation in the chloroplast: many features of the prokaryotic translation mechanism are retained in the chloroplast (Zoschke & Bock, 2018) and, extending on previous observations (Masuda *et al*., 2008b; Masuda, 2012), we can identify chloroplast orthologues of all the major bacterial (p)ppGpp‐targeted enzymes involved in translation regulation (Fig. 3c; Table 1).

Despite the promising theoretical situation, there is still relatively little experimental evidence on the effects of (p)ppGpp on chloroplast translation. Using *an in vitro* chloroplast translation system from pea (*Pisum sativum*), ppGpp was found to inhibit the peptide elongation cycle of chloroplast translation by *c*. 50% at 400 μM (Nomura *et al*., 2012). This is consistent with the presence of LepA (Ji *et al*., 2012) and SNOWY COTYLEDON1 (Albrecht *et al*., 2006) in the chloroplast, orthologues of EF4 and EF‐G which participate in polypeptide elongation in bacteria and are well‐known targets of inhibition by (p)ppGpp (Fig. 3c; Table 1) (Bange *et al*., 2021). Artificial accumulation of ppGpp *in vivo* was also found to have a major effect on chloroplast translation, as measured by the incorporation of the antibiotic puromycin (a structural analogue of aminoacyl‐tRNA) into nascent peptide chains (Sugliani *et al*., 2016). However, the observed inhibition was difficult to separate from the transcriptional downregulation of rRNA and tRNA that is also caused by ppGpp.

Recently, the role of (p)ppGpp in promoting the stress‐induced hibernation of bacterial ribosomes has received particular attention (Prossliner *et al*., 2018; Trösch & Willmund, 2019; Irving *et al*., 2020; Bange *et al*., 2021). Under stress conditions, bacterial 70S ribosomes are inactivated as monomers or dimers that are also known as 100S ribosomes. Inactivation contributes to the downregulation of translation and also allows rapid re‐activation of translation upon return to favourable conditions. Notably, (p)ppGpp accumulation promotes the transcriptional upregulation of hibernation factors such as ribosome‐associated inhibitor A, ribosome modulation factor and hibernation promoting factor (HPF) that trigger ribosome inactivation. The chloroplasts of plants and algae possess an HPF orthologue named plastid‐specific ribosomal protein 1 (PSRP1, Fig. 3c) that can trigger the formation of inactive 70S monomers (Sharma *et al*., 2010). However, the physiological function of PSRP1 is not yet elucidated (Swift *et al*., 2020), and it is unlikely to be transcriptionally regulated by (p)ppGpp as it is encoded on the nuclear genome. The ribosome‐associated GTPase high frequency of lysogeny X (HflX) is also implicated in ribosome inactivation in bacteria. In *Staphylococcus aureus*, HflX is able to dissociate the hibernating 100S complex and this activity is inhibited by (p)ppGpp binding (Basu & Yap, 2017). Hflx and other GTPases involved in ribosome biogenesis and assembly (RsgA, RbgA, Era, Obg) are all inhibited by (p)ppGpp to reduce subunit maturation or prevent 70S assembly in the translation cycle (Bennison *et al*., 2019). Notably, and as discussed earlier, Obg and its chloroplast orthologue ObgC share conserved interactions with RSH enzymes (Wout *et al*., 2004; Bang *et al*., 2012; Chen *et al*., 2014), indicating that there is a profound link between (p)ppGpp signalling and ribosome biogenesis that appears to have been maintained over a vast expanse of evolutionary time.

### 4. Are there chloroplast‐specific targets of (p)ppGpp signalling?

Since the original acquisition of the chloroplast, there has been ample time for the evolution of new (p)ppGpp signalling mechanisms. Furthermore, the cohabitation of the chloroplast and the eukaryotic cell, the development of multicellularity and the colonisation of new niches including the land would have provided powerful selection pressures to drive the emergence of novel mechanisms. Chloroplastic GTPases are prime candidates as ppGpp targets simply because ppGpp has a tendency to target GTPases in bacteria (Fig. 3c; Table 1). Outside translation, only a handful of chloroplast GTPases are known, and these play roles in ribosome assembly, photosynthesis, chloroplast division, vesicle trafficking and membrane remodelling. The circularly permuted GTPases SUPPRESSOR OF VARIEGATION 10 and BRZ INSENSITIVE PALE GREEN2 are implicated in chloroplast ribosome assembly (Qi *et al*., 2016); the GTPase PsbO is a subunit of the oxygen evolving complex involved in the turnover of the PSII reaction centre (Spetea *et al*., 2004; Lundin *et al*., 2007); chloroplast FtsZ tubulin‐like GTPases ensure the formation of a contractile ring within the stroma during chloroplast division (Osteryoung & Vierling, 1995; Yoshida *et al*., 2016); the chloroplast‐localised Rab family small GTPases are implicated in chloroplast vesicle trafficking (Ebine *et al*., 2011; Alezzawi, 2014; Alezzawi *et al*., 2014; Karim *et al*., 2014; Karim & Aronsson, 2014); and finally the GTPase vesicle‐inducing protein in plastids 1 (VIPP1) is essential for the biogenesis and maintenance of thylakoid membranes (Ohnishi *et al*., 2018; Gupta *et al*., 2021). Interestingly, accumulation of ppGpp was shown to cause hyper‐stacking of the thylakoid membranes in the moss *Physcomitrium patens* (Harchouni *et al*., 2022). While this might simply be explained by an increase in the quantity of PSII antenna subunits, it also raises the possibility that ppGpp can promote membrane remodelling by acting on proteins like VIPP1. Beyond the GTPases, there may be evidence that other classes of protein are targeted by ppGpp. For example, we previously speculated that the higher sensitivity of some chloroplast proteins to ppGpp, and Rubisco in particular, could involve the ppGpp‐mediated regulation of chloroplast protein turnover (Romand *et al*., 2022). However, it is clear that more intensive studies aimed specifically at identifying chloroplast (p)ppGpp targets using both candidate‐based and more open‐ended approaches will be required to move beyond speculation.

### 5. A larger family of related signalling nucleotides in plants?

In this review, we have dealt exclusively with (p)ppGpp. However, these are just two of a larger family of related nucleotides that also include pGpp and (p)ppApp. Discovered in the 1970s (Oki *et al*., 1976; Nishino *et al*., 1979), pGpp and (p)ppApp can be synthesised by RSH and SAS enzymes, and their functions are stimulating renewed interest among microbiologists. pGpp, ppGpp and pppGpp act in similar ways, though have preferences for different target enzymes (Gaca *et al*., 2015; Yang *et al*., 2020b). (p)ppApp, on the other hand, can bind RNAP on a different site to (p)ppGpp and is able to activate transcription (Travers, 1978; Bruhn‐Olszewska *et al*., 2018). To our knowledge, there are no reports of the detection of pGpp or (p)ppApp in plants or algae. Furthermore, while ppGpp is now readily detected (Ihara *et al*., 2015; Jin *et al*., 2018; Bartoli *et al*., 2020), pppGpp has only been reported once (Takahashi *et al*., 2004) suggesting that it is unstable or present only under certain circumstances. In future, it will be interesting to determine whether other members of the extended (p)ppGpp family of nucleotides are present in plants and algae, and what role they play.

## V. Concluding remarks

(p)ppGpp was originally discovered > 50 yr ago (Cashel & Gallant, 1969). Since that time, work on bacteria, algae, plants and more recently animals has revealed the extraordinary diversity and reach of (p)ppGpp signalling. Over recent years, our understanding of (p)ppGpp signalling in plants and algae has advanced considerably, thanks to studies of its physiological roles *in vivo* notably revealing the conserved action of (p)ppGpp on photosynthesis and its likely role in regulating cellular carbon/nitrogen status. To understand how (p)ppGpp acts at a molecular level, it will be necessary to build on the early *in vitro* experiments and adopt new approaches including those so successfully employed in bacteria for identifying the physiologically relevant targets of (p)ppGpp and related nucleotides. Likewise, the functional diversification of plant and algal members of the RSH superfamily promises to reveal exciting new features of (p)ppGpp signalling.
